# Clinical Practice Recommendations on the Routine Use of Eversense, the First Long-Term Implantable Continuous Glucose Monitoring System

**DOI:** 10.1089/dia.2018.0397

**Published:** 2019-05-07

**Authors:** Dorothee Deiss, Agnieszka Szadkowska, Debbie Gordon, Akhila Mallipedhi, Ingrid Schütz-Fuhrmann, Eva Aguilera, Corina Ringsell, Christophe De Block, Concetta Irace

**Affiliations:** ^1^ Center for Endocrinology and Diabetology, Medicover Berlin-Mitte, Berlin, Germany.; ^2^ Department of Pediatrics, Diabetology, Endocrinology and Nephrology, Medical University of Lodz, Łódź, Poland.; ^3^ Center for Diabetes and Endocrinology, Johannesburg, South Africa.; ^4^ Donald Gordon Medical Centre, WITS (University of the Witwatersrand), Johannesburg, South Africa.; ^5^ Hywel Dda University Health Board, Llanelli, United Kingdom.; ^6^ Division of Endocrinology, Third Department for Internal Medicine, City-Hospital Hietzing Vienna, Vienna, Austria.; ^7^ Department of Endocrinology and Nutrition, CIBER of Diabetes and Associated Metabolic Diseases, Health Sciences Research Institute and University Hospital Germans Trias i Pujol, Badalona, Spain.; ^8^ Universimed Holding GmbH, Medical Writing, Vienna, Austria.; ^9^ Department of Endocrinology, Diabetology and Metabolism, Antwerp University Hospital, University of Antwerp, Antwerp, Belgium.; ^10^ Department of Health Science, University Magna Græcia, Catanzaro, Italy.

**Keywords:** Clinical practice guide, Long-term implantable glucose monitoring, Long-term continuous glucose monitoring system, Real-time continuous glucose monitoring system

## Abstract

***Background:*** The use of real-time continuous glucose monitoring (rtCGM) systems has proved to positively impact the management of type 1 diabetes with the potential to lower HbA1c, reduce frequency and time spent in hypoglycemia, and lower glycemic variability. Nevertheless, the acceptance of rtCGM remains below expectations and the dropout rate within the first year has been reported to be 27%. Besides financial reasons due to limited reimbursement, reasons include the need for frequent sensor replacement, the discomfort of wearing a sensor, the presence of adverse skin reactions, or privacy. Thus, novel approaches to rtCGM are desired to overcome these barriers. The first long-term implantable rtCGM system diversifies the field of glucose monitoring further. However, due to its novelty, there are no published clinical practice guidelines available.

***Aims:*** The aim of this article is to set the foundation for a best clinical practice for the everyday clinical care using a long-term implantable CGM system.

***Methods:*** An international expert panel for the long-term implantable CGM system developed this best practice guidance. All participants were certified and experienced in the use of the Eversense^®^ long-term implantable CGM system. The workflows from the respective clinics were presented, discussed and are summarized in an ideal care workflow outlined in these practice recommendations.

***Results:*** The participants agreed on the following aspects: definition of the patient population that will benefit from a long-term implantable CGM device; real-world experience on safety and accuracy of a long-term CGM; definition of the ideal sensor position; description of the optimal process for sensor insertion, removal, and replacement.

## Introduction

Extensive evidence highlights that real-time continuous glucose monitoring (rtCGM) improves glycemic control and quality of life in both children and adults with type 1 diabetes (T1D) treated with continuous subcutaneous insulin infusion or multiple daily insulin injection therapy.^1–3^ Improvements include lowering of HbA1c, reduction of time spent in hypoglycemia and hyperglycemia, increased time spent in range and lowering the incidence rate of moderate-to-severe hypoglycemia.^4–11^ rtCGM use in pregnant women with T1D is associated with improved neonatal outcomes.^12^

Despite the highlighted benefits, CGM usage among patients with T1D is still limited with even poorer uptake among patients with type 2 diabetes (T2D).^13–16^ In addition to financial reasons due to limited reimbursement, reasons for CGMs limited use include a perceived burden of frequent insertions, fear of pain or discomfort, the likelihood of accidental sensor dislocation, potential for drug interferences, privacy reasons, and the occurrence of skin reactions to the adhesive.^17–20^

In addition, CGM discontinuation rate is high with 27% of users terminating the therapy and an even higher number of users showing limited adherence to the therapy within the first year for similar reasons as mentioned above.^20–22^ Thus, to fully realize the benefits of rtCGM-supported therapy, further development of continuous glucose monitor devices is needed.

The first implantable long-term rtCGM (LTI rtCGM) has the tradename Eversense^®^ (Senseonics, Maryland) and is the only implantable glucose monitoring technology currently available on the market. This system extends the possible average CGM wear time from 7–14 days to up to 180 days. The Eversense system received CE Marking in 2016 with approved use for up to 90 days. Subsequently, the product use time was extended for up to 180 days, and this product (Eversense XL) received CE Marking in 2017. In the United States, the Eversense system received U.S. Food and Drug Administration (FDA) approval for use up to 90 days in June of 2018.^23,24^

This rtCGM system consists of a subcutaneously inserted sensor, a wearable transmitter, and a smartphone application. The sensor longevity is achieved using multiple approaches, including the chemical structure of the glucose binding polymer, the platinum coating, the dexamethasone acetate eluting ring, and the subcutaneous position in comparison to transcutaneous rtCGM systems.^24^

Unlike transcutaneous CGM (TC CGM) systems where the patient does self-insertion, the Eversense system requires professional placement by a health care provider. While a comprehensive procedure training program is provided by the manufacturer, the sharing of experiences among health care providers is also necessary to help advance the use of this novel technology.^25^

As endocrinologists from Europe and South Africa not involved in any of the pivotal studies, we provide a first attempt to reflect and share our experience with the LTI rtCGM system in the clinical routine setting. All off-label practices with respect to the long-term implantable CGM described below were undertaken under our responsibility, independent of the manufacturer and sponsor of this article. Neither the manufacturer (Senseonics, Inc.) nor the local distributor (Roche Diabetes Care) promoted or encouraged these practices, nor requested their inclusion in this article. The decision to provide information on off-label practices was undertaken solely by the authors in conjunction with the peer review of the article. With the awareness that more implantable CGM systems are on the horizon, this practice recommendation based on our experiences with the Eversense CGM system can also aid future implantable glucose systems. This article especially aims to provide information on the following: (1) indications and patient selection, (2) how to insert and remove the sensor, and (3) how to handle the follow-up of the patients after the procedures.

## Current Indications for CGM-Based Therapy

Clinical indications for the use of CGM are well defined and target two major challenges in diabetes management: avoiding hypoglycemia and achieving personal treatment goals.^26–30^ The detailed clinical patient stratification between rtCGM and intermittently scanned CGM system (isCGM), also known as flash glucose monitoring, has been discussed elsewhere.^26,30^

Alongside the clinical indication, the patient's preference of and commitment to the chosen medical device are essential determinates for the success of any therapy. Detailed patient information and the evaluation of the patient's willingness and capability to perform the required measures to benefit from a CGM are prerequisites.

One such aspect that is often perceived as an additional burden is CGM calibration since it involves the need for patients to test blood glucose level within a certain window of time.^31^ While calibration is required by some CGM systems at least two times per day,^32^ the factory calibration of the Dexcom G6 (Dexcom, CA) and the Freestyle Libre (Abbott, CA) systems makes regular calibration in those systems either optional or unnecessary.^33,34^

However, from our experience, calibration is a means of quality assurance of the accuracy of the CGM sensor over time and from batch to batch in respect to an individual's requirements. Therefore, optional calibration by the patient might improve accuracy of the individual sensor during its wear. The patient needs to be fully aware of the specific concept of calibration of his CGM system to assess sensor accuracy over time.

### Patient selection for LTI rtCGM

**FIG. 1.**
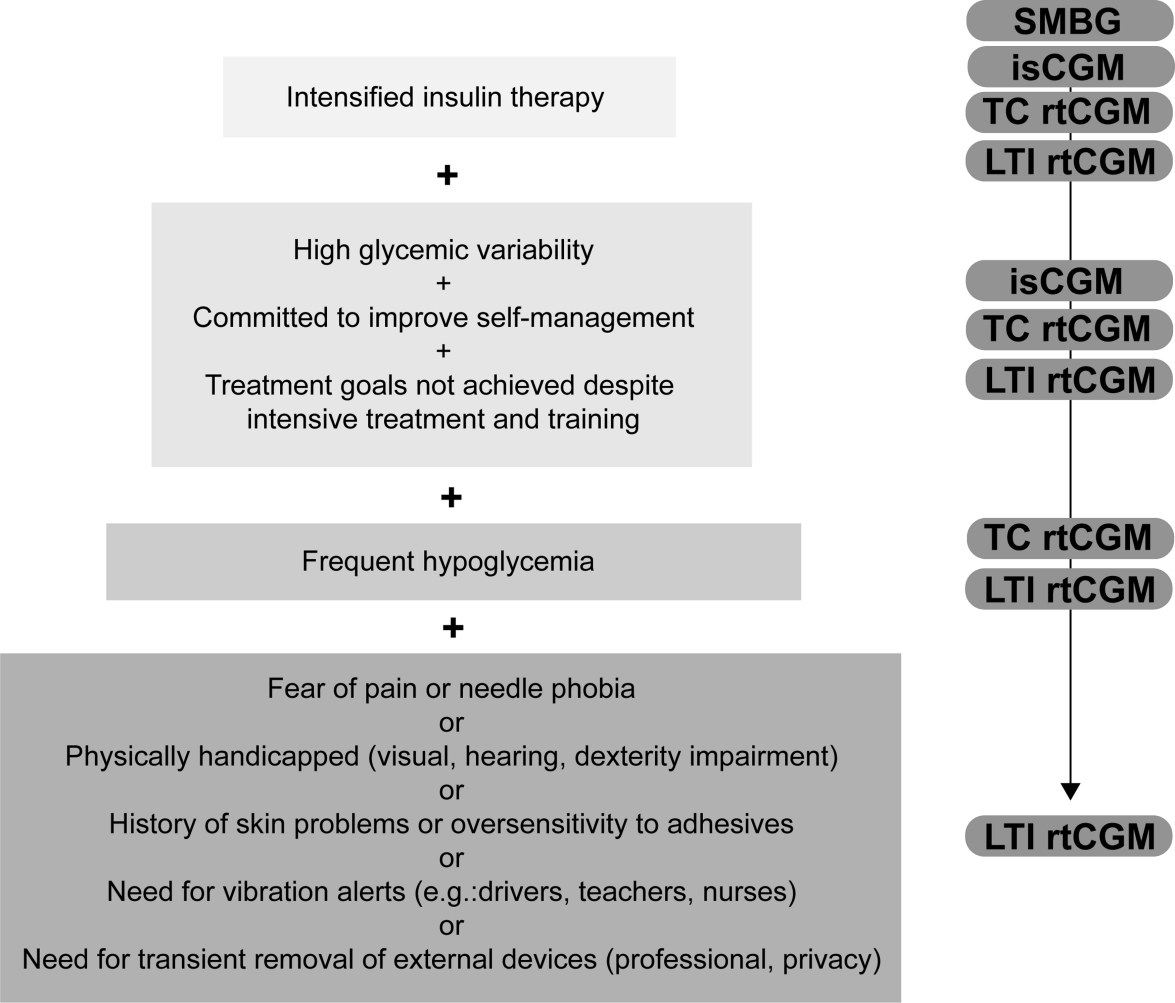
Decision tree for glucose monitoring. Selection criteria for the optimal glucose monitoring device. Intensified insulin users have a wide choice of glucose monitoring devices according to their personal preference. With addition of further clinical indications or lifestyle restrictions from top to bottom, the use of specific devices is recommended. isCGM, intermittent scanning continuous glucose monitoring; LTI rtCGM, long-term implantable real-time CGM; SMBG, self-monitoring of capillary blood glucose; TC rtCGM, transcutaneous real-time CGM.

In addition to all generally applicable CGM selection criteria as mentioned above, further specific characteristics are suggested to identify those patients who would benefit most from a LTI rtCGM (Fig. 1):

- People who perceive frequent sensor replacement as a burden
- Reasons for patients refusing weekly or biweekly sensor replacement can range from logistical hurdles and inconvenience to pain, discomfort, and needle phobia. Furthermore, people with special physical needs or visual impairment, for whom the self-application is a barrier, could also benefit from provider-based sensor insertions.
- People who benefit from on-body vibration alerts
- The LTI rtCGM provides customized low- and high-glucose alerts, including predictive low- and high-glucose warnings. Specifically, the patient is alerted via their smartphone app as well as from the wearable Eversense transmitter. As the glucose calculation is performed in the transmitter, it is the transmitter that provides the specific alert using distinct vibratory patterns independent of the smartphone app.
- This vibratory alert thus may provide unique advantages for some patients, including those individuals who are restricted in their ability to view their smartphone when an alert condition is triggered. This could be due to their employment restrictions such as those specific to professional drivers, athletes (esp. swimmers), manual laborers, nurses, or teachers. This can also be driven by lifestyle and a desire for privacy during important events such as a business meeting or a job interview, during events that require full cognition such as driving, and even during other private situations such as intimacy. It also includes those with visual and/or hearing impairment and those who would not be woken up by audible alarms while sleeping.
- People with isobornyl acrylate oversensitivity:
- Recent studies highlight that exposure to isobornyl acrylate over an extended period of time (more than 4 days) can cause severe skin reactions such as redness, itching, or pain and in severe cases, to those who are susceptible, can cause an allergic immune response.^35,36^ As most adhesives for CGM systems are based on isobornyl acrylate, this affects the usability of CGM and can even result in the need to stop using the device in some cases.^35^ Thus, any patient with a history of skin problems or allergies to adhesives could benefit from the isobornyl acrylate-free adhesive used for holding the Eversense transmitter in place.
- People with the need for the flexibility to remove externally attached devices:
- Since the wearable transmitter of the system may be removed and replaced without disturbing the sensor, this may be of interest for those active or work-constrained individuals, including athletic people participating in contact sports, people who work in clean rooms and laboratories with a high sterility degree, people at enhanced risk of accidentally pulling off transcutaneous sensors, or any other occupation which would prevent the use of externally attached devices.

Additionally, the ability to remove the transmitter and change the adhesive daily allows for routine skincare and can be of help in preventing skin reactions.

- People for whom a permanent breach of the natural skin barrier cannot be advised. This would include individuals exposed to extensive levels of dust due to their profession or lifestyle.

According to our clinical experience, most patients who started on the Eversense CGM continue to use the system. The longest LTI CGM system adherence span so far has been patients who are on their 10th sensor at the moment. Reasons for terminating the therapy form, which happens only in a very low percentage, include insufficient initial information and training and inappropriate expectations about rtCGM in general.

The Eversense user manual includes specific situations or patient groups to be contraindicated or not studied.^24^ We report our clinical experience on several of those topics:

- A scheduled magnetic resonance imaging (MRI) during the sensor wear time: In several of our patients an unforeseeable MRI needed to be done while a LTI rtCGM was in place. In these cases, no adverse event (AE) occurred and all sensors remained functional after the MRI procedure. Furthermore, the Eversense has recently obtained FDA indication for MRI use.*
- The need for intravenous mannitol or sorbitol irrigation or tetracyclines. To date, we have not gained experience on this point.
- Known contraindication to dexamethasone. The total dexamethasone content of <2 mg is similar to the dose used for dual chamber pacing systems, which proved to be safe.^37^ To date, we have not seen any patient with contraindication to dexamethasone.
- Children and adolescents younger than 18 years of age: first insights about the use of Eversense in adolescents between 12 and 17 years of age were gained in Canada and were presented at the ADA 2018. In this open-label study, 36 participants were included, of which 83% were younger than 18 years of age.^38^ While the authors did not show any statistically significant evidence that sensor longevity or accuracy was influenced by age, the strength of the exploratory analysis was constrained by low statistical power. Furthermore, a single-arm study is currently underway in Germany to evaluate the use of Eversense in the pediatric population from 6 to 18 years of age.^39^

## Prerequisites for CGM Initiation

### Patient education

Our experience with CGM highlights the importance of diligent patient information and education before starting therapy with any CGM device to foster therapy adherence and patient satisfaction. Tailored patient education is obligatory before initiation of any CGM supported therapy. The optimal training encompasses three parts, which include the principles of sensor technology, the operational aspects of the device, and the interpretation of the derived data. This initial training is ideally scheduled 1 week before using the system, and in most cases, technically skilled patients or CGM-experienced patients will acquire the necessary training in a single session. It is also important to check phone compatibility for CGM mobile application before insertion. For centers with limited experience or resources, there are commercially available patient education curricula and easy-to-use trend arrow recommendations in form of scorecards, which give a detailed guide on educational essentials for CGM users.^40–42^

In the special case of CGM naive users, a minimal set of glucose alerts should be programmed in the beginning. This avoids alarm fatigue that might overwhelm a new user. Using a stepwise approach, rtCGM naive users should start with the most important alert functions (such as hypoglycemia alerts). Nevertheless, after a period of 3–4 weeks, the patient should aim to use all essential alert functions (hypo, hyper, predictive low, and predictive high).

The only aspect specific to the LTI rtCGM device is that the training should also cover the topic of incision care after the insertion and removal procedures.

A comprehensive patient education on CGM in the beginning of their experience will enable patients to review the glucose data regularly and make informed decisions resulting in a sustainable benefit in glucose control. Insufficient patient information before starting CGM-supported therapy is a major driver for low CGM retention. This holds especially true for the LTI rtCGM system, for which a trial period is not possible.

### Patient information and written consent

The insertion of the LTI rtCGM sensor is a minimally invasive procedure, which requires informing the patient of all related benefits and risks. Therefore, the patient may have to sign an informed consent before the procedure for the clinic's records. The consent should encompass all aspects of a minimally invasive procedure and templates can be obtained from different sources such as national medical societies or Senseonics, Inc. (https://ous.eversensediabetes.com/healthcare-providers). A brief description would include evaluation of potential allergies to lidocaine or dexamethasone, detailed information regarding the procedure, recommended incision care, and a detailed description of possible AEs.

## Insertion and Removal of the Eversense Sensor

Structured as a stepwise instruction manual, this section details the recommended best clinical practice.

The following general prerequisites should be considered:

- Any physician interested in undertaking the procedure will be accompanied and consequently certified by the company's clinical training manager (CTM) during the first several insertions and removals to ensure the required training to comply with the highest quality standards.
- The patient should be able to lie down during the procedure.
- No sterile procedure room is required, but high hygiene standards such as clean clothes, sterile surgical gloves, and disinfected surfaces form the basis of a successful procedure. A face mask is advisable in case the physician is having a severe cold or cough to prevent infections.
- The procedure is ideally performed cooperatively by a trained health care provider and an assistant to divide up the sterile and nonsterile responsibilities.
- Optimization of the setup such as using a quiet room free of external disturbances and ensuring practical aspects are thought through such as creating minimal distances between the patient, sterile field, and waste bin, while still ensuring functional separation, guarantees a tension-free atmosphere.
- A written protocol of the procedure of sensor insertion and removal should be readily available in the native language of the physician and the assistant.

### The sensor placement

For the sensor insertion, a duration of 20–30 min should be scheduled for all preparation steps, patient information, and the insertion itself. The placement of the sensor itself, from making the incision to closing it, takes 3–5 min.

Since the location of the sensor predefines the location of the transmitter, deciding on the exact position of the transmitter on the upper arm together with the patient will enhance patient satisfaction and foster therapy adherence. Thus, the first step is to define the position of the device on the upper arm of the patient taking the personal preference into account.

We advise placing the first sensor in the nondominant arm (e.g., left arm in right-handed patients) to ease the daily positioning of the transmitter. This rule can be critically evaluated if any specific habit of the patient (including sleeping position, dominant arm in sports, shoulder bag wearing, or frequency of car driving, which increases the risk for ambient light alerts on the side of the window) would suggest an alternative prioritization. Deciding on the position of the sensor on the chosen arm should be done in an upright body position, while the muscles are tensed for the physician to feel the borders of the relevant arm muscles.

From our experience, the best results are archived if the system is placed in the fossa one finger wide of the caudal boarder of the deltoid muscle, posterior of the brachialis and anterior of the triceps muscle parallel to the humerus bone. While the instructions state to place the sensor midway between the acromion and the lateral epicondyle, we have found that with respect to the bone structure, the optimal location for the sensor insertion coincided with 1/3 caudal of the acromion and 2/3 cranial from the articulatio cubiti of the whole length of the humerus and posterior and in parallel of the humerus bone.

**FIG. 2.**
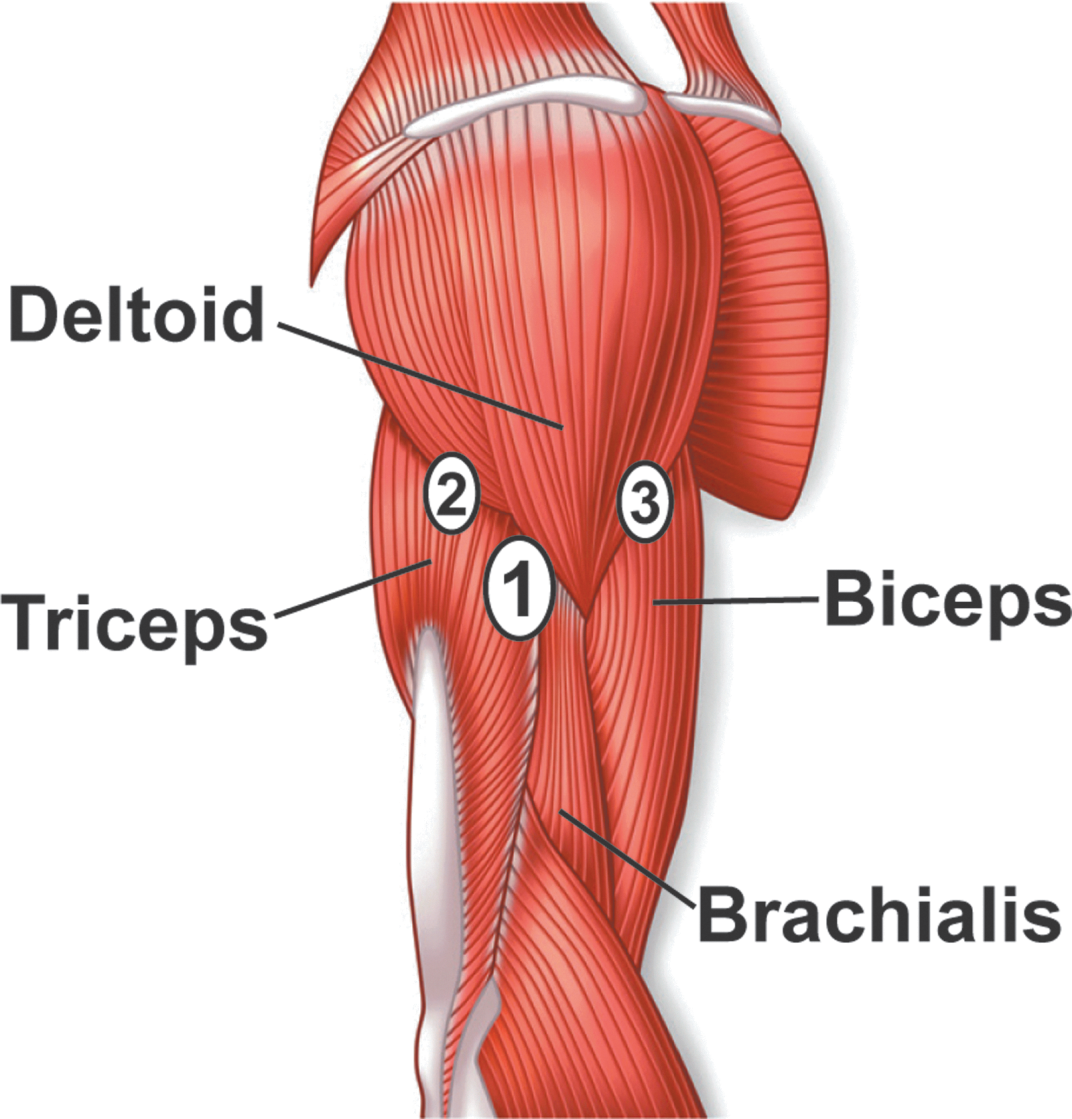
Anatomic schema of positioning the sensor. If no preference is indicated, the ideal location for the sensor insertion is the fossa between the triceps and the brachialis one finger wide caudal of the deltoid muscle (1: primary site). According to personal preferences, the insertion site can also be further posterior (2: alternative site) or further anterior (3: alternative site) along the caudal edge of the deltoid muscle.

In some cases, the patient asks for a position more anterior (predominantly adipose individuals) or posterior (predominantly slim individuals) along the caudal border of the deltoid muscle, which has also shown to give good results. Especially in trained individuals, we would not advise positioning the sensor in the subcutis directly on top of one of the upper arm muscles, since this might enhance the induced tension on the system and could foster sensor dislocation or signal loss of the transmitter (Fig. 2).^25,37^

The approved site for the Eversense CGM system is the upper arm. Sensor placements in alternative sites as an off-label procedure performed by some of us were exclusively done according to the wish of the patient and included abdomen and buttock. Our experience shows that all sensors placed in alternative sites met the accuracy and usability demands of the individual patient and no particular AE occurred due to placing the sensor elsewhere than the upper arm. For all areas of the body where the subcutaneous fat layer is expected to be thicker and its tone to be lower than on the upper arm, the insertion as well as the removal procedure is more demanding on the physician than the respective procedure on the upper arm.

**FIG. 3.**
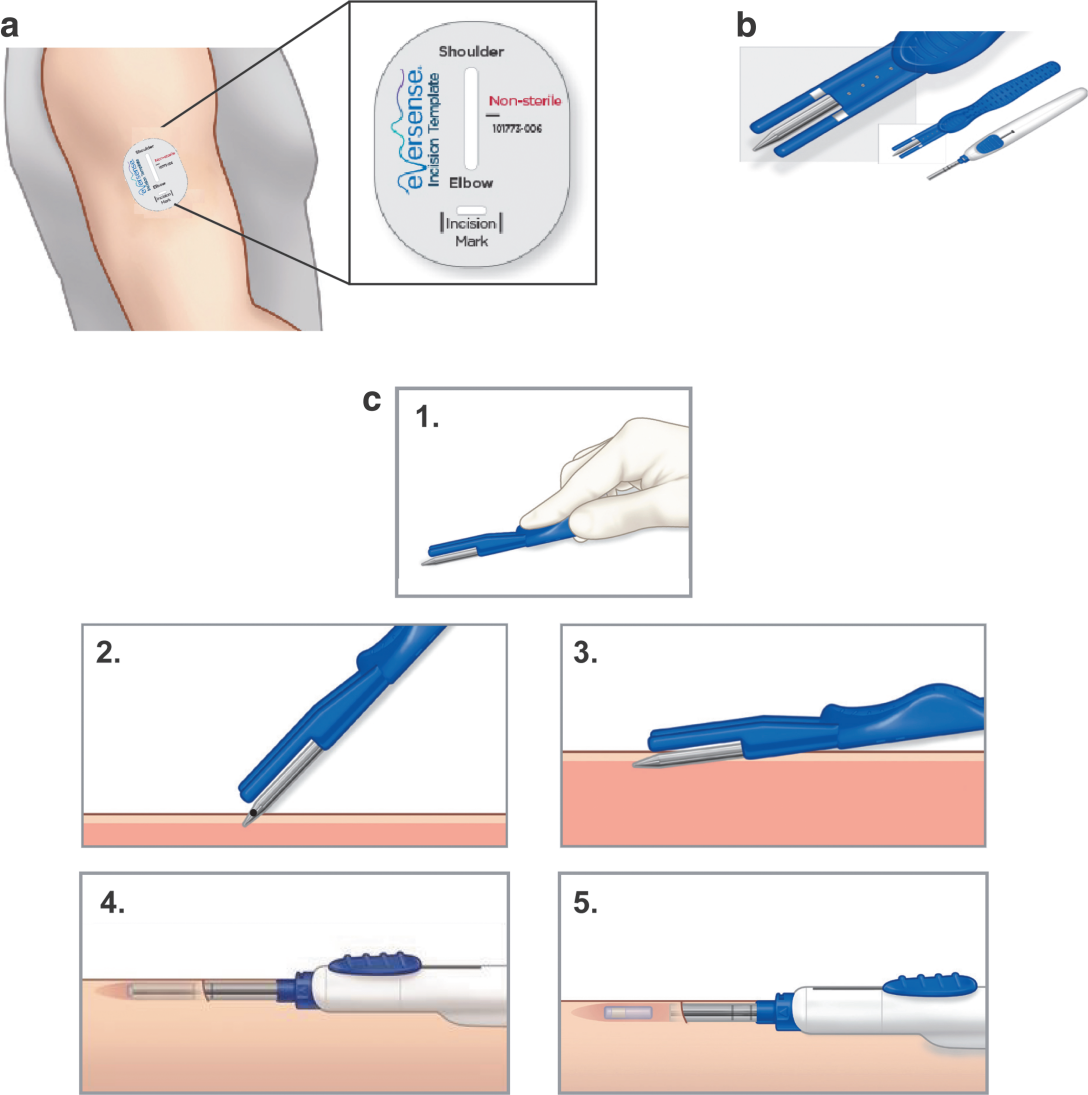
Eversense sensor placement. **(a)** The positioning guide for the Eversense system is used to determine the location of the sensor according to the chosen position for the transmitter. The horizontal recess indicates the incision site and the vertical recess the location of the subcutaneous pocket for the sensor. **(b)** Blunt dissector and insertion tool. The blunt dissector (blue handle) exhibits depth guards on either side of the dissector. The insertion tool (white handle) is used to position the sensor in the subcutaneous pocket. **(c)** Sensor insertion procedure. Description of the procedure in five steps—1. Depicts the correct handling of the blunt dissector. The index finger is pushed into the deepening on the top of the device while the remaining fingers hold the device from above. 2. The blunt dissector is introduced into the incision. 3. The blunt dissector is advanced into the subcutaneous tissue parallel to the skin surface. 4. The insertion device holding the preloaded sensor is inserted into the newly formed subcutaneous pocket. 5. The slider on the insertion tool is retracted and the sensor is disposed into the subcutaneous pocket.

The manufacturer's incision template (Fig. 3a) is used to mark the location of the sensor with respect to the location the patient chooses to wear the transmitter. Most important is the location of the sensor with respect to the skin surface which will determine the effort needed to remove the sensor after its wear time. Ideally, sensors are placed parallel to and right underneath the dermal layer of the skin (at a depth of ∼3–5 mm), which places it in the subcutaneous tissue. A newly designed blunt dissector with two depth guards ensures shallow sensor placement and has recently become available for the routine use (Fig. 3b).

The sensor itself is cylindrical in form and measures 3.5 × 18.3 mm. It is inserted according to the following steps (Fig. 3c)^25^:

- The physician prepares for the aseptic procedure by cleaning all surfaces, washing hands, and setting up the sterile field.
- The patient lies on his/her side with the chosen arm positioned slightly angled and fully relaxed on top of the torso.
- The insertion tool (white handle) is hydrated with sterile saline solution. The sensor is loaded into the tool, and then returned to a 10 mL well of sterile saline solution in the designated device tray to hydrate for 5 min before it is inserted.
- The insertion site is cleaned and disinfected using three passes of the disinfectant (starting at the planned insertion site and moving circularly outwards, waiting 30 s between each pass) and draped.
- Lidocaine (1%–2%) is sterilely drawn with the help of the assistant and injected along the planned incision site and the line indicating the future position of the sensor, using the smallest required volume (∼1.5 mL).
- A 5 mm incision is made at the previously marked insertion site (horizontal line of the incision template) once anesthesia is in effect. Our experience showed that the use of the scalpel size 15 allows for the smallest possible incision to be made. Apply gentle pressure on the incision site to control any bleeding and minimize potential bruising.
- A subcutaneous pocket is formed by inserting the blunt dissector (blue tool) into the subcutaneous tissue at a depth of ∼3–5 mm. Following the vertical line that was drawn using the incision template, the pocket is formed parallel to the humerus bone. Holding the dissector with the fingers on top and on the side of the device and not underneath of it ensures a shallow sensor placement (Fig. 2-3). Slight rotation of the blunt dissector around its center axis will allow the tool to advance through potential tissue resistance. If desired, the dissector can be kept in the newly formed pocket for a few seconds while applying gentle pressure from on top before removing it.
- The hydrated sensor is inserted into the subcutaneous pocket using the insertion tool (white tool) (Fig. 3b–c). After inserting, apply gentle pressure for several minutes to achieve hemostasis and minimize bruising.
- The incision site is cleaned and dried before closing it with Steri-Strips™. Sutures are not recommended to minimize scarring.
- A sterile, waterproof dressing is applied on top of the Steri-Strips to keep the incision clean and dry.

After the insertion, the sensor and smart transmitter are linked by placing the transmitter over the sensor. This will start the 24 h warm-up phase, during which the transmitter is not worn and no glucose readings are displayed.^24,25^

On the first day following the first 24 h warm-up phase, the system requires two calibrations to display the first glucose readings. The system will ask for the first calibration right after the 24 h warm-up phase. The second one can be done 2 h later, which will result in the first glucose readings being displayed ∼26 h after insertion of the sensor. Two additional calibrations are requested from the system on day 1 (total of four calibrations between 24 and 48 h after insertion), which will further refine the sensor accuracy. We recommend performing the first two calibrations as soon as possible after the warm-up phase, to start the active use of the sensor.

It takes 5–7 days in total to maximize the strength of the incision across its full length.^43^ During this time, the transmitter is attached on top of the waterproof adhesive dressing.

For an optimal incision healing, the following rules apply:

- No extensive strain on the arm for at least 72 h.
- No extensive arm muscle work out for 5 days.
- No swimming, soaking the arm, or sauna for 5 days.
- Steri-Strips should stay in place at least 5 days. Allow them to fall off on their own.
- A waterproof adhesive dressing should be worn on top of the Steri-Strips. The dressing should be changed if it gets soiled and should remain in place until Steri-Strips have fallen off.
- Showers are allowed if the stream of water is directed away from the incision.

### The sensor removal

The duration of a sensor removal procedure will on average take up to 20–30 min, including all preparation steps, patient information, and the actual procedure. This period can vary and depends on the experience of the physician, the location of the sensor underneath the skin, and the structure of the subcutaneous tissue of the individual patient. Since predicting the exact duration of the procedure is therefore difficult, we suggest scheduling the removal appointment with a flexible time window (e.g., at the end of clinic appointment schedule). In our experience, the strongest determinant of the time to remove the sensor is the quality of the sensor placement, and even though we have faced a few more demanding cases, no patient so far has terminated the Eversense use due to a complicated removal process.

The following workflow for the removal of the sensor should be in line with the following rules:

- Removal attempts should only be made if the physician is able to palpate the sensor or see the incision line from the insertion. If neither one of these landmarks is available, the physician should only proceed after identifying the sensor's location with ultrasound.
- With the patient positioned on his/her side for the removal, the patient's smart transmitter and placement guide in the app should be used to mark the approximate location of the sensor. Use senses of sight and touch to locate the sensor while the arm is fully relaxed. As soon as the sensor is localized, the distal and proximal ends of the sensor are held with two fingers, and the sensor is marked using a skin marker pen. Also mark the incision line. If the sensor's distal end is within 3 mm of the original incision line, consider using the same line for the removal. If the original incision line is either not visible or more than 3 mm away from the sensor's distal end, mark a new incision line for the removal, which is 1–2 mm below the sensor's distal end.

The sensor is removed according to the following steps:

- The patient lies on his/her side with the arm positioned slightly angled and fully relaxed on top of the torso.
- Incision site is cleaned, disinfected, and draped. Be sure that syringe with lidocaine, scalpel, and small straight surgical clamp with serrated surface are within reach.
- Stabilize the sensor's proximal end with one finger of the nondominant hand during the entire procedure to simplify the removal process.
- Anesthesia using injectable lidocaine is done identically to the insertion procedure. Limiting the volume of anesthetic to the absolute minimum, which is generally ∼1–1.5 mL, minimizes unwanted tissue swelling and difficulties of palpating the sensor.
- A 5–6 mm incision is made using a scalpel size 15.
- A small straight surgical clamp with serrated surface in its unlocked but closed position is inserted into the incision and is advanced until the distal end of the sensor is reached. Make sure to not apply any pressure at this point to prevent accidentally pushing the sensor further into the pocket. The clamp is opened and the sensor is positioned in the jaws of the clamps. The exact alignment of the clamp and the sensor allows smooth extraction of the sensor.
- Use your tactile sense while moving the clamp to localize the sensor underneath the skin.
- Be aware that you are working in the three-dimensional space and that the sensor might be right above or below the clamp.
- Slight rotational motion can help to free the sensor from potential encapsulations.
- By pulling the clamp with the sensor out of the incision, the sensor is removed.
- The incision is closed with Steri-Strips, and a sterile waterproof dressing is applied.
- Incision care is identical to the measures taken following sensor insertion.
- The quality of the procedure and the end result for the patient increase with greater experience and routine in performing the procedure. Thus, taking the initial training seriously and collaborating with the CTM in cases of difficulty is strongly advised.

### The sensor replacement

After the first sensor insertion, sensor removal and insertion of the subsequent sensor can be done in one appointment. According to the manufacturer, the subsequent sensor is placed into a subcutaneous pocket on the alternative arm to allow healing and avoid any undue trauma to the area.

While the label of Eversense recommends that the same incision or pocket is not used for consecutive sensor insertions, we recognize that there might be circumstances that dictate use of the same incision line and/or subcutaneous pocket. Our experience, while limited, shows that it can be a burden for the patient to insert each sensor in alternating arms. Therefore, most of us have used the same pocket to place a new 90-day sensor, as long as the removal from this pocket was uncomplicated.

Since the wear time for the Eversense system was extended to up to 180 days, we have still reused the same incision line when possible, but always formed a new subcutaneous pocket in a minimum of 45° from the previously used subcutaneous pocket. From our experience, the number of possible sensor replacements using the same incision depends on the individual's scar formation. In case the same incision cannot be used, a novel incision is to be made not less than 5 mm away from the original incision due to tissue vascularization concerns.

## Potential Adverse Events (AEs) of rtCGM Systems

Potential AEs for rtCGM range from skin reactions such as tape allergies to scars, infections, inaccurate measurements, preterm sensor failure, or accidental sensor dislocation. These are described in great detail elsewhere.^19,22,35,44^ Pivotal studies for the Eversense were undertaken to clearly document potential unwanted side effects.^23,37^

The accuracy and safety of the Eversense system were shown in patients with either T1D or T2D in the PRECISE study in Europe and the PRECISE II and the PRECISION studies in the United States.^23,25,37^

Eversense users in Europe who were tracked across four sensors had a wear time above 90%, which remained stable over the entire study period.^45^

Evidence on the incidence rate of any AEs obtained in the pivotal trials PRECISE (*n* = 71; 292 skin incisions), PRECISE II (*n* = 90; 212 skin incisions), and PRECISION study (*n* = 35; 124 skin incisions) was shown to be low.^23,25,37^ The low rate of AEs is consistent with what is seen in the comprehensive European registry with all Eversense users enrolled since 2016.^25^

Since starting the work with the LTI rtCGM system, we observed the following incidences:

- Temporary skin thinning at the site of the sensor, which can in rare cases persist for an extended period of time (>3 months) after sensor removal. We hypothesize that this could be induced by the eluted amount of dexamethasone acetate from the silicone rubber ring, which amounts on average to 3 μg dexamethasone per day over the life of the sensor, to minimize the local innate inflammatory response.^24^ These cases exhibit similarities to tissue atrophy observed in other long-term glucocorticoid treatments where full restoration of the tissue can take up to 3 years, but has been observed in all cases studied.^46^ Due to the lower amount of glucocorticoid concentration by an order of magnitude eluted from the Eversense system than the concentration in previous reports, we expect a faster and complete restoration in all cases.
- Prolonged wound healing (longer than 5–7 days) in rare cases, mostly caused by insufficient wound care.
- Temporary blue discoloring or depigmentation of the skin at the sensor site, which disappears after the sensor has been removed.
- Preterm sensor failure. Like any other CGM, the sensor life might not last through its entirety. While from our experience a large portion of patients have had the sensor last up to 180 days, we have also seen a few cases whose sensor ended before 180 days. Additional real-world evidence is needed to quantitatively verify the longevity data of the Eversense from the most recent clinical study presented at the ADA 2018, which indicates a 78% sensors survival at 180 days wear time.^38^
- Intermittent unavailability of data. In our experience this can have two major causes. The first being loss of connection between the sensor and the transmitter. This can be minimized by following the transmitter's positioning function on the app. The second reason is related to an “ambient light” alert, which mainly occur within the first 7 days of wear time. In this case, extensive ambient light can stop the sensor from measuring glucose temporarily. This can be resolved by shielding the area of the transmitter against external light.
- Difficulties removing the sensor. By following the above suggested process to locate the sensor before removal using diligent palpation, stabilizing the sensor throughout the whole procedure with one-finger technique and finally to use ultrasound, if needed, has resolved all difficult cases.
- We did not experience any loss of sensor parts under the skin. In the PRECISE II study, a few cases were observed. These AEs were rated as mild in severity due to the small size of the element and biocompatibility of the material.^37^
- Hypersensitivity to lidocaine. To date neither the pivotal trials nor our clinical experience have reported any such adverse drug reactions. Cases of hypersensitivity to lidocaine causing skin irritations, urticaria, bronchospasm, angioedema, psychomotor responses as vasovagal attack, and hyperventilation or, in severe cases, anaphylactic shock have been reported as rare in the literature (<1/100) and usually occur after exposure to much higher doses of local anesthetics (LA).
- Both immediate (type 1) and delayed (type 4) hypersensitivity reactions can occur, but the generally reported incidence of IgE-mediated allergy is low and remains <1% in subjects with suspected LA allergy.^47^ Accidental intravascular injection should be avoided by using safe injection techniques (aspiring prior injection). Potential allergy to lidocaine should be further assessed if clinical patient history shows indications, using skin testing and a challenge if appropriate.^47^

### How to minimize AEs

#### Monitoring and preventing AEs at regular visits

Similar to the use of a transdermal rtCGM system, to identify potential device-related, procedure-related, or drug-related (dexamethasone acetate) events, the attending physician should examine all insertion and removal sites at any follow-up visit and look for the potential presence of early sensor failure, skin irritation at the site of the adhesive patch, including redness, excoriation or ulceration, pain or discomfort, and redness or infection at the site of the sensor.

In case any of these symptoms or situations occurs, we suggest selecting the other arm for the insertion of the replacement sensor.

Especially when experience is limited, the incision site should be examined 1 week after sensor placement or removal, and any findings should be documented with standardized photography. With increased experience, this follow-up can often be done using a telephone checkup. We recommend that incisions are examined and documented at each regular visit and before removal/reinsertion of the sensor.

#### Handling of any potential AEs during sensor wear time

To recognize and resolve any AE, the user, the health care provider, and the company's customer service need to work together closely.

As soon as a patient needs advice on a potential AE, we advise our patients to take pictures of any sign and send those to his/her diabetes team. Furthermore, the patient has to inform the customer service of any problem as soon as it occurs. To date, we have not seen any case, which would have made the removal of a functioning sensor necessary. In case of technical issues, taking screenshots of error messages on the smartphone screen eases the problem-solving process. It should be clear to the patient for which issues he should contact his/her own doctor (e.g., clinical manifestations) and for which ones he/she needs to inform customer support (technical failure).

#### Measures to prevent potential AEs during sensor removal

Highest precision of the insertion and smallest possible quantity of injectable lidocaine will minimize the probability of complications during the removal procedure.

In case the sensor cannot be localized or removed within 30 min, we suggest the following:

- Close the incision and allow it to heal for about 48 h. Reschedule the second attempt for when a colleague with more foreign body removal experience and the company CTM are available.
- The CTM can be requested from the manufacturer, who will aid the physician to successfully locate and remove the sensor. To date, no participant of this panel has faced the need of involving a surgeon, which would only enhance unnecessary logistic complexity and psychological burden for the patient.

## Comparing the LTI rtCGM System to TC CGM Systems

When comparing technical aspects and usability of TC CGM systems to the LTI CGMs, the following differences exist:

- The LTI CGM eliminates the need for weekly sensor changes and insertions.
- The long sensor life of up to 180 days reduces the frequency of lower-accuracy day-1 glucose readings of TC CGM. The LTI CGM has a warm-up period of 24 h, in which the sensor does not display any glucose values. Subsequently, the sensor is calibrated four times during the first active day of wear. With a wear time of up to 180 days, the warm-up and initial calibration procedure have to be undertaken on average two to three times per year. Similar to other CGM systems, the initial lower accuracy state can last potentially from 1 day to 1 week after sensor initiation.
- The LTI system requires two calibrations daily in contrast to some TC systems, for which daily calibration is not necessary.
- The problem of unwanted sensor dislocation becomes obsolete. However, in case of a rare preterm sensor failure, the replacement has to be scheduled with the Health Care Professional (HCP) and might expose the patient to a short time period without CGM data.
- Due to the position of the LTI rtCGM system underneath the skin, patients should be aware that “trial” insertions for a very limited time (a couple of days) are not advisable. Thus, patients in doubt of the applicability of the CGM technology to their lifestyle should gain experience using a short-term CGM system first, for example, isCGM should subsequently switch to the LTI rtCGM device once convinced that the technology is right for them.
- Adhesives for TC CGMs, which by necessity are in place for 6- to 10-fold longer, require a much stronger bond to the skin to keep the sensor in place, which has caused skin irritations. In contrast to that, the adhesive of the LTI CGMs system is silicone-based and is changed daily, which aids the prevention of skin reactions.
- The first head to head accuracy study including the Eversense system was presented at the ADA annual meeting in 2018.^48^ This three-way comparison study analyzed the accuracy of the Eversense, Dexcom G5, and Libre Pro in an outpatient study of subjects with T1D and highlighted the MARDs to be 14.8%, 16.3%, and 18.0%, respectively.^48^
- The LTI CGMs minimizes the influence of human factors such as misplaced TC sensors by the patients. However, to insert and remove the LTI CGM sensor, health care providers need to have adequate training and routine.
- Compression-induced underestimation of glucose concentration is not present in LTI CGM. However, if the transmitter moves on the skin during sleep, signal loss can occur.
- The transmitter in the LTI CGM is removable and water- and dust-resistant (IP67). It can be taken off and put on at the patient's discretion. The transmitter needs to be recharged at least every 30 h, which has to be consciously integrated in the patient's daily routine.
- In addition to common smartphone app alarms, the LTI rtCGM system gives alarms in form of vibration patterns directly on the skin, with no need of having the smartphone or device display in proximity.

## Summary

The first commercially available long-term implantable rtCGM system targets some of the most frequent causes for the limited CGM penetration and long-term adherence. It provides enhanced skin tolerance, long-term accuracy, the freedom of choice to remove any external parts when needed, and additional safety with on-body vibration alerts.

Due to the novelty of the system, this device and the corresponding procedure are just now being introduced into the clinical endocrinology routine. Even though the required minimally invasive procedure exceeds the area of experience for many diabetologists at first sight, the technique is easy to learn when performed with proper training, oversight, and attention to detail. These practice recommendations give a general overview on aspects such as which patients will benefit most from this novel technology and a detailed guide for placing and removing the long-term implantable sensor.

## Authors' Contributions

All authors contributed to the interpretation of the collected literature and in the development of the content; all authors and Roche reviewed and approved the article; the authors maintained control over the final content.

## Notes

^*^ FDA has approved that a patient with this device can be safely scanned in an MRI system meeting the following conditions: static magnetic field of 1.5 and 3.0 T; maximum spatial field gradient of 2000 gauss/cm (20 T/m); and maximum MRI system reported, whole body averaged specific absorption rate of 4 W/kg (First Level Controlled Operating Mode).
